# Association of *TLR4* Polymorphisms, Expression, and Vitamin D with *Helicobacter pylori* Infection

**DOI:** 10.3390/jpm9010002

**Published:** 2019-01-11

**Authors:** Shafika Assaad, Christy Costanian, Lama Jaffal, Fida Tannous, Maria G. Stathopoulou, Said El Shamieh

**Affiliations:** 1 Department of Life and Earth Sciences, Faculty of Sciences II, Lebanese University, Fanar P.O. Box 26110217, Lebanon; cassaad@ul.edu.lb; 2 Faculty of Medicine, University of Ottawa, Ottawa, ON K1G 5Z3, Canada; christycostanian@gmail.com; 3 Rammal Hassan Rammal Research Laboratory, Physio-toxicity (PhyTox) research group, Lebanese University, Faculty of Sciences (V), Nabatieh, Lebanon; lama.jaffal66@gmail.com; 4 Department of Biological and Environmental Sciences, Faculty of Science, Beirut Arab University, Lebanon; fida.tannous@hotmail.com; 5 Université de Lorraine, Inserm, IGE-PCV, F-54000 Nancy, France; maria.stathopoulou@inserm.fr; 6 Department of Medical Laboratory Technology, Faculty of Health Sciences, Beirut Arab University, Beirut 1107 2809, Lebanon

**Keywords:** *Helicobacter pylori*, *Toll-like Receptor 4*, single nucleotide polymorphisms, gene expression, vitamin D

## Abstract

*Helicobacter pylori* (*H. pylori*) infection is the strongest recognized risk factor for gastric adenocarcinoma. Since previous observations have shown that polymorphisms in innate immune system genes, as well as vitamin D (VitD) levels, could modify the risk of infection with *Helicobacter* *pylori* (*H. pylori*), we analyzed the relation between single nucleotide polymorphisms (SNPs) in *TLRs* (*TLR1*, *TLR2*, *TLR4*) *CD14*, *RUNX3* and VitD levels with *H. pylori* infection. A case-control study on four hundred sixty Lebanese individuals was conducted. Eleven SNPs in total were genotyped and gene expression analysis using real-time PCR was performed in white blood cells of a subsample of eight individuals. A total of 49% of the participants were affected. Although no direct association was found between the SNPs and *H. pylori* infection, rs4986790G>A and rs4986791T>C in *TLR4* were negatively associated with VitD levels (β = −0.371, *p* = 5 × 10^−3^ and β = −0.4, *p* = 2 × 10^−3^, respectively), which was negatively associated with *H. pylori* infection (OR = 0.01, *p* < 1 × 10^−3^). *TLR4* expression was 3× lower in individuals with *H. pylori* compared with non-infected (*p* = 0.01). *TLR4* polymorphisms, expression, and VitD could be implicated in *H. pylori* infection and further development of gastric adenocarcinoma.

## 1. Introduction

*Helicobacter pylori* (*H. pylori*) infection has high incidence rates worldwide and is the major cause of several gastrointestinal symptoms, ranging from mild gastritis to gastric adenocarcinoma [1]. It has been estimated that up to half of the world’s population harbor this infection in their stomach [1]. Population-based studies report that developing countries have a higher prevalence rate of *H. pylori* than their developed counterparts [1]. Using data from a nationally representative, cross-sectional study on Lebanese adults, Naja et al. found that the prevalence of *H. pylori* infection was 52%, a rate comparable to other developing countries [2].

The host innate immune system plays a key role in the initiation and subsequent progression of *H. pylori*-associated pathogenesis [3]. The reason for the variable phenotypic expression of the infection is multifactorial and combines the effects of bacterial virulence factors, host genetic constitution, and environmental exposures [4]. Gastric epithelial cells are the primary target and the first point of contact for *H. pylori* infection, which actively contribute to the innate immune responses by signaling through pattern recognition receptors, such as toll-like receptors (TLRs) [4]. One of the factors that could modify *H. pylori* infection is genetic predisposition; single nucleotide polymorphisms (SNPs) in *TLR* genes, such as *TLR1*, *TLR2*, *TLR4* and *TLR10*, were shown to alter the bacterial binding, thereby modulating the risk of infection and subsequent cancer [5]. A large genome-wide association study (GWAS) meta-analysis, conducted on 10,938 individuals, identified an association between rs10004195 in *TLR1* and *H. pylori* seroprevalence [6]. In addition, many other small-scale studies revealed that SNPs, in genes such as *TLR2*, *TLR4*, *CD14* and *RUNX3*, are associated with *H. pylori* infection and/or seropositivity [6,7,8,9,10]. Despite their importance, these findings should be investigated among other independent populations [6].

In addition to the innate immune system, serum vitamin D (VitD) levels have been implicated in *H. pylori* infection in the literature in recent years [11]. Low VitD concentration could be a predisposing factor for severe Th1-type aggression to the stomach epithelium in *H. pylori* gastritis patients [11]. The proposed mechanism for this link is via VitD-inducing immune modulator properties against *H. pylori* by decreasing inflammatory chemokine and cytokine levels [12]. In fact, it was shown that VitD production in macrophages is stimulated by *TLRs* as part of the innate immune response to intracellular bacteria [13,14].

Based on all the above, the association between polymorphisms in *TLRs CD14*, *RUNX3* and VitD with *H. pylori* infection warrants further investigation. Therefore, we investigated the association between eleven SNPs (Supplementary Data, Table S1) and VitD with *H. pylori* infection in a Lebanese population composed of four hundred sixty individuals, divided between cases and controls. Furthermore, we assessed the gene expression of *TLR4* in white blood cells (WBCs) of a subsample of eight individuals divided according to *H. pylori* infection status.

## 2. Methods

### 2.1. Study Design and Participants

This was a cross-sectional population-based study involving four hundred sixty Lebanese unrelated individuals free of chronic disease (cardiovascular disease or cancer). Recruitment occurred between 2015 and 2016 at the Hospital center of North-Zgharta a major tertiary care hospital in Northern Lebanon. Study participants aged 18 years or above were recruited at the gastroenterological unit after being referred for endoscopic examination of the upper gastrointestinal (GI) tract (gastroscopy) to obtain a biopsy. A retrospective chart review was conducted to determine study eligibility. Only patients with GI (dyspeptic) symptoms, mainly epigastric pain and gastritis, and who were undergoing gastroscopy were included in the study. *H. pylori* status (positive or negative) was determined after upper GI endoscopy, where gastric biopsy specimens from the antrum, body, and fundus region were collected on a plate containing formalin buffer. A pathologist examined these samples using hematoxylin and eosin (H&E) staining. The semi-quantitative method of scoring according to the Updated Sydney Classification System was applied. Controls were first examined through the same procedure and not found to be carrying *H. pylori*; in addition, they were matched in terms of age, gender, body mass index (BMI) categories, marital status and alcohol consumption.

### 2.2. Data Collection

Participants involved in the present study were recruited in accordance with the latest version of the Declaration of Helsinki for Ethical Principles for Medical Research Involving Human Subjects. Ethical approval was obtained from the local institutional review board Clinical Research Ethics Committee at the Lebanese University. Protocols for genetic studies were approved by the local ethics committees for the protection of subjects involved in biomedical research (2182/28). We obtained written informed consent from all the participants.

Complete medical examinations were carried out for all individuals, with data collected including demographic, anthropometric, clinical and measurements. A BMI was considered normal when a value of < 25 kg/m^2^ was observed. Biochemical measurements including glycemia, lipid profile, and VitD were measured with commercial kits (Roche Diagnostics, Basel, Switzerland). Serum levels of total 25-hydroxyvitamin D [25(OH) D], which is the sum of D2 and D3, were measured using Elecsys™ Vitamin D total assay (Roche Diagnostics, Basel, Switzerland). Vitamin D deficiency and insufficiency were described for levels of 25(OH)D falling below 30 ng/mL and below 20 ng/mL, respectively [15].

### 2.3. Genomic DNA Extraction and Genotyping Assays

DNA extraction was performed on whole blood samples obtained from all participants, using a DNA extraction kit from Qiagen (QIAamp DNA Mini Kit, Hilden, Germany), according to the manufacturer’s protocol. A Qubit 3.0 fluorometer (Thermo Fisher Scientific, Selangor, Malaysia) was used to quantify the DNA extracts using the Qubit dsDNA BR Assay Kit (Thermo Fisher Scientific, Malaysia). We selected eleven SNPs for genotyping (Supplementary Data, Table S1). Genotyping was performed by the LGC group (Berlin, Germany) using KASP as described previously [16]. 

### 2.4. TLR4 Expression in White Blood Cells

Total RNA was isolated from WBCs by an automated isolation procedure using an Aurum™ Total RNA Mini Kit (Life Science Research, Bio-Rad, Singapore, Singapore). RNA quality and stability were carefully tested on 1% agarose gel and reverse-transcribed using an iScript™ cDNA Synthesis Kit (Life Science Research, Bio-Rad). We used designed specific primers for *TLR4* quantification using Primer3 software, version 0.4.0 (data available upon request). We carried out cDNA amplification in a reaction volume of 20 uL consisting of 70 ng of cDNA, Solis BioDyne 1× HOT FIREPol Blend Master Mix (Tartu, Estonia) and both primers. Quantitative real-time PCR (qRT-PCR) was performed using the CFX Connect™ Real-Time PCR Detection System (Bio-Rad) with an iTaq Universal SYBR Green Supermix Kit (Bio-Rad) for *TLR4* transcripts. All experiments were carried out in duplicates in a total reaction volume of 20 µL containing 0.5 mM of each specific primer. Negative and internal controls were included. All mRNA levels were normalized to the mRNA levels of *RNA polymerase II subunit A* (*POLR2A*). The specificity of all PCR products was further verified by electrophoresis on 3% polyacrylamide gel.

### 2.5. Statistical Analyses

Statistical analyses were carried out using SPSS software, version 20.0 (SPSS, Inc., Chicago, IL, USA). Demographic characteristics (age, gender) anthropometric characteristics (height and weight), clinical (systolic and diastolic blood pressure) and biochemical measurements (lipid profile, glucose, VitD) were compared according to *H. pylori* infection using chi-square tests for categorical variables and t-tests for continuous ones. Normality was tested using the Shapiro–Wilk test and, when needed, continuous variables were log-transformed to improve their normality. SNPs (rs4833095 in *TLR2*) deviating from the Hardy–Weinberg equilibrium or showing a null MAF (rs5743708 in *TLR2*) were excluded.

A multivariate logistic regression was performed to first ascertain the association between all SNPs and *H. pylori* infection in a model adjusted for age, gender, BMI, education level, marital status, alcohol consumption, and VitD status. Next, multivariate linear regression was performed for all SNPs and VitD levels. All regression analyses (using three models of inheritance: Additive, recessive and dominant) were adjusted for age and gender. The significance level was set at *p* ≤ 0.005 due to multiple testing.

A Mann–Whitney U-test was performed to compare *TLR4* expression levels in the WBCs of *H. pylori* patients (*n* = 5), compared to controls (*n* = 3). The significance level was set at *p* < 0.05.

## 3. Results

The demographic, clinical, and biochemical characteristics of participants are shown in Table 1. Approximately half of our participants (49%) were found to have *H. pylori* infection. Whereas the majority of variables matched between cases and controls, some significant differences were noticed (Table 1). The most apparent significant difference between the two groups was serum VitD levels (*p* = 1 × 10^−4^, Table 2). Individuals infected with *H. pylori* were VitD-deficient, whereas non-affected individuals had normal VitD levels (*p* < 1 × 10^−3^, Table 1). In addition to VitD, educational level varied significantly between cases and controls, revealing that the highest infection rate is among those with a university degree (Table 1).

Gene polymorphisms were not associated with *H. pylori* when adjusted for different confounding factors (*p* > 5 × 10^−3^, Table 2). Among the dependent variables, we found that, while having a university degree increases the risk of *H. pylori* (OR = 4.16, *p* = 0.01), normal VitD levels decrease it remarkably (OR = 0.01, *p* < 1 × 10^−3^, Table 2).

On the other hand, rs4986790G>A and rs4986791T>C in *TLR4* were negatively associated with VitD levels (Table 3). Thus, we concluded that TLR4 SNPs are associated with decreased VitD levels, which could lead to an increased risk of *H. pylori* infection.

**Figure 1:**
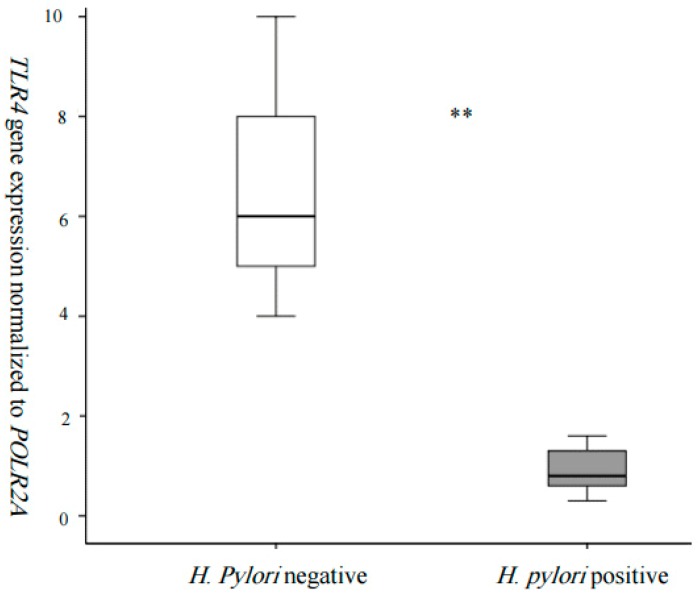
*TLR4* expression in whole blood cells according to *H. pylori* infection (** *p* < 0.05).

To better assess the relation between *TLR4* and *H. pylori*, we decided to study its expression in the WBCs of a subsample of individuals who were stratified according to their infection status. Gene expression analysis showed that *TLR4* was 3 times lower in the WBCs of *H. pylori*-affected individuals compared to non-affected (Figure 1).

Quantitative real-time PCR, normalized to the expression of *POLR2A*, revealed 3× higher expression in the WBCs of with *H. pylori* (*n* = 3) when compared to *H. pylori* (*n* = 5) (** *p* < 0.01).

## 4. Discussion

Herein, we found that rs4986790G>A and rs4986791T>C in *TLR4* were associated with VitD, which in turn affected *H. pylori* infection, implying that those SNPs could have an indirect effect on *H. pylori*. Specifically, individuals with *H. pylori* were found to be VitD-deficient. Finally, *TLR4* expression was shown to be 3× lower in the WBCs of *H. pylori* individuals, compared to unaffected ones.

The prevalence of *H. pylori* infection in this study was found to be 49%, which is comparable to the general Lebanese adult population, according to Naja et al. [2]. This rate is lower than that found in other countries of the Middle East and North Africa region, including Egypt, Libya, Saudi Arabia, Iran, Oman, United Arab Emirates, and Turkey, where the prevalence of *H. pylori* is higher [14].

We chose to study rs10004195 in *TLR1* since it is known to be associated with *H. pylori* seroprevalence [6]. Despite its importance, we were unable to replicate rs10004195, which can be explained in a number of ways: Compared to Mayerle et al., our sample was highly limited, which makes replication of SNPs with small effects difficult; in contrast to Europeans, Lebanese individuals are multiethnic, originating from less homogeneous descendants, meaning that association studies can be challenging. 

The significant negative association between VitD and *H. pylori* in our study is in concordance with recent findings in previous STUDES [17,18,19]. Yildirim et al. found in their study that VitD deficiency could be a risk factor related to the eradication failure of *H. pylori*, suggesting the need for the supplementation of VitD before the eradication of *H. pylori* [19]. In a cross-sectional study, Nasri and Baradaran reported that VitD potentiates the immune response of dialysis patients to *H. pylori* and could positively affect their chronic inflammatory status [18]. This study suggested that the analogs of VitD, in addition to its antibacterial action against *H. pylori*, might offer patients on maintenance dialysis a new means by which to control their inflammatory status [18]. Interestingly, previous studies did indicate the antibacterial effects of VitD. According to Hosoda et al., the antibacterial action of VitD against *H. pylori* is linked to the VitD3 decomposition product, which induces a collapse of cell membrane structures of *H. pylori* and ultimately lyses the bacterial cells [17]. Recently, Wanibuchi et al. revealed that indene compounds synthetically derived from VitD perform selective antibacterial action against *H. pylori* [20]. Consequently, VitD3 is suggested as a fundamental structure for the development of new antibacterial substances, providing selective bactericidal action against *H. pylori* [17].

Since VitD is known for its antineoplastic and antioxidant properties, several studies have explored its relation to cancer progression [21]. In fact, it is involved in cell cycle regulation, cellular proliferation, apoptosis, and angiogenesis [22]. Growing evidence is showing that deficiency in VitD is associated with an increased risk of gastric adenocarcinoma and a poor prognosis [23]. Recently, Singh et al. reported that low serum VitD levels may play a role in the development of incomplete intestinal metaplasia, which is the most frequently observed precancerous change in the gastric mucosa [21].

Existing data show that the total number of WBCs and the numbers of lymphocytes and basophils were significantly increased in *H. pylori*-positive individuals, compared with unaffected ones [24]. In fact, Kondo et al. demonstrated that the eradication of *H. pylori* decreases blood neutrophil and monocyte counts [25]. These observations highlight the importance of choosing WBCs as a model to study the relation between *TLR4* and *H. pylori*. Gene expression analysis showed that *TLR4* was 3 times lower in the WBCs of *H. pylori*-affected individuals, compared to controls (Figure 1). Despite the fact that *TLR4* expression varied significantly in the WBCs of *H. pylori* individuals, compared to controls, the difference in expression was modest. This implies that TLR4 is not the sole receptor implicated in *H*. *pylori* recognition and the subsequent host response. Evidence for TLR4 implication in *H. pylori* infection is still contradictory, while Ishara et al. [26] demonstrated that *H. pylori* infection upregulates the host’s innate immunity through the activation of the TLR4–MD-2 system in the gastric epithelial cells [26]. Smith et al. [27] demonstrated that infection from *H. pylori* induces responses via TLR2 and TLR5, but surprisingly not TLR4 [27]. This is in contrast with previous studies on mice and guinea pigs, which have provided evidence that TLR4 may be an important contributing factor to the inflammatory response induced by *H. pylori* [28]. In the current study, we found a modest change in *TLR4* expression which may not be detected at the protein levels, because it can be masked by a post-transcriptional control mechanism (such as miRNAs). All the above underlines the necessity of working on adequate cell models to study gene expression when using high-throughput sequencing techniques.

Our findings point out that *TLR4* expression in monocytes was altered by the effect of VitD [29]. Supporting our results, in vitro, Do et al. demonstrated that VitD3 was able to modify the protein and mRNA expression of *TLR4* [30]. These observations support the hypothesis that VitD may act as an immune modulator. In this regard, it is not surprising to find that *TLR4* expression decreased in the WBCs of *H. pylori* individuals, since they were shown to have low VitD levels, an event that predisposed them to *H. pylori* infection.

When compared to previous studies, our goal was broader, since we did not only focus on VitD, but we expanded the associations to study the genetics of *TLRs* and other risk factors in a multiethnic population (Lebanese population). Adding gene expression data in WBCs that are reported to be altered in *H. pylori*-positive individuals is of interest. Our study has several limitations. The study sample was limited to adult patients at a tertiary healthcare center in one region of Lebanon; therefore, our results cannot be generalized. Our gene expression analysis was done on a quite limited sample size and, thus, it is preliminary, normally, thirty samples are needed to have reasonable and conclusive results.

In conclusion, no direct association was found between *TLRs* and *H. pylori* infection; by contrast, SNPs in *TLR4* were negatively associated with serum VitD levels, which was negatively associated with *H. pylori* infection. Finally, *TLR4* expression differed according to *H. pylori* infection. All of the above point to a possible implication in *H. pylori* infection and further development of gastric adenocarcinoma.

## Figures and Tables

**Table 1:** Biological characteristics of the study population according to *Helicobacter pylori* infection.

	*Helicobacter pylori*		
	Positive (*n* = 225)	Negative (*n* = 235)	*p*
	Mean ± SD	Mean ± SD	
**Age (years)**	39.28 ± 13.9	41.86 ± 14.26	0.05
**Gender**			
Males, *n* (%)	88 (39.1%)	80 (34%)	0.25
Females, *n* (%)	137 (60.9%)	155 (66%)	
**BMI Category**			
Normal	109	128	0.12
Overweight and Obese	116	107	
**Education Level**			
Primary	32	56	6 × 10^−3^
School	52	57	
University	141	122	
**Marital Status**			
Not Married	70	69	0.38
Married	155	166	
**Alcohol consumption**			
No	145	153	0.48
Yes	80	82	
**SBP (mmHg)**	131.03 ± 1.51	131.37 ± 1.64	0.05
**DBP (mmHg)**	6.78 ± 0.88	6.78 ± 0.93	0.47
**Glycemia (mg/dL)**	121 ± 46	95 ± 21	0.18
**Cholesterol (mmol/L)**	1.75 ± 0.41	1.87 ± 0.4	0.009
**TG (mmol/L)**	1.22 ± 0.92	1.68 ± 1.45	0.001
**HDL (mmol/L)**	0.46 ± 0.12	0.44 ± 0.16	0.13
**LDL (mmol/L)**	1.14 ± 0.34	1.2 ± 0.32	0.24
**VitD (ng/mL)**	18.04 ± 7.16	30.74 ± 15.66	<1 × 10^−3^
Insufficiency	133	12	<1 × 10^−3^
Deficiency	56	109	
Normal	15	107	

Values are arithmetic mean ± standard deviation (SD) or proportion (%). BMI, body mass index; SBP, systolic blood pressure; DBP, diastolic blood pressure; TG, triglycerides; HDL, high-density lipoproteins; LDL, low-density lipoproteins; VitD, vitamin D.

**Table 2:** Multiple logistic regression analysis of different risk factors with *Helicobacter pylori* infection.

	*Helicobacter pylori*		
	OR	95% C.I.	*p*
**Age**			
<40 years	1		
≥40 years	0.73	(0.33–1.62)	0.44
**Gender**			
Male	1		
Female	0.7	(0.37–1.32)	0.02
**BMI category**			
Normal	1		
Overweight and Obese	1.49	(0.82–2.72)	0.19
**Education Level**			
Primary	1		
School	2.46	(0.96–6.34)	0.06
University	4.16	(1.52–11.4)	0.01
**Marital Status**			
Not Married	1		
Married	1.03	(0.5–2.1)	0.93
**Alcohol Consumption**			
No	1		
Yes	1.42	(0.76–2.67)	0.27
**Vitamin D status**			
Insufficiency	1		
Deficiency	0.03	(0.01–0.06)	<1 × 10^−3^
Normal	0.01	(0–0.02)	<1 × 10^−3^
**Gene SNPs**			
rs10004195 in *TLR1*	1.23	(0.8–1.8)	0.29
rs10759932 in *TLR4*	1.37	(0.75–2.48)	0.30
rs10983755 in *TLR4*	0.51	(0.05–5.44)	0.58
rs11536889 in *TLR4*	1.51	(0.82–2.79)	0.19
rs1898830 in *TLR4*	0.84	(0.55–1.29)	0.44
rs4986790 in *TLR4*	7.09	(1.12–45)	0.04
rs4986791 in *TLR4*	0.14	(0.02–0.86)	0.03
rs2569190 in *CD14*	0.74	(0.49–1.12)	0.15
rs760805 in *RUNX3*	0.78	(0.53–1.15)	0.21

Reference categories of gene single nucleotide polymorphisms were not shown since no significant association was found. C.I., confidence interval.

**Table 3:** Association of rs4986790G>A and rs4986791T>C in *TLR4* with serum vitamin D levels.

Vitamin D							
SNP ID	Gene	Chr	position	*n*	β [95% C.I.]	S.E	*p*
rs4986790G>A	*TLR4*	9	117713024	456	−0.371 [−0.112, −0.63]	0.13	5 × 10^−3^
rs4986791T>C			117713324	457	−0.4 [−0.14, −0.663]	0.13	1 × 10^−3^

Chr: Chromosome, *n*: Sample size, S.E., standard error.

## Supplementary Materials

The following are available online at http://www.mdpi.com/2075-4426/9/1/2/s1, Table S1: List of studies single nucleotide polymorphisms, Table S2: Minor allele frequency of single nucleotide polymorphisms, Table S3: Analysis of deviation from Hardy Weinberg equilibrium.
